# A Functional Shape Framework for the Detection of Multiple Sclerosis Using Optical Coherence Tomography Images

**DOI:** 10.3390/s26082399

**Published:** 2026-04-14

**Authors:** Homa Tahvilian, Raheleh Kafieh, Fereshteh Ashtari, M. N. S. Swamy, M. Omair Ahmad

**Affiliations:** 1 Department of Electrical and Computer Engineering, Concordia University, Montréal, QC H3G 1M8, Canada; homa.tahvilian@mail.concordia.ca (H.T.); swamy@ece.concordia.ca (M.N.S.S.); 2 Department of Engineering, Durham University, Durham DH1 3LE, UK; raheleh.kafieh@durham.ac.uk; 3 Isfahan Neurosciences Research Center, Isfahan University of Medical Sciences, Isfahan 81746-73461, Iran; f_ashtari@med.mui.ac.ir

**Keywords:** multiple sclerosis, optical coherence tomography, atlas registration, functional shape, support vector machine classifier

## Abstract

Multiple sclerosis (MS) is an inflammatory and neurodegenerative disease. Optical coherence tomography (OCT) is a non-invasive imaging technique of the retina. The thickness of the ganglion cell–inner plexiform layer (GCIPL) obtained from an OCT image is a valuable biomarker for monitoring MS. Since the functional shape (F-shape)-based technique has proven to be an effective platform for detecting glaucoma using OCT images, in this paper, we develop an F-shape-based framework to distinguish MS subjects from healthy ones using the thickness of GCIPL. The thickness of the GCIPL layers in the macula region of OCT images in a selected region of interest (ROI) for a set of healthy and MS subjects is represented as F-shape objects, which are registered to a common template using atlas registration. The residual F-shapes, defined as the difference between the F-shape of this common template and the individual registered F-shapes, are used to train an support vector machine (SVM) classifier and subsequently to detect MS. Accuracy, sensitivity, specificity, and area under the curve (AUC) are used to evaluate and compare the classification performance of the proposed F-shape-based scheme and those of sectoral-based schemes. The proposed F-shape-based scheme is shown to significantly outperform the sectoral-based schemes. The superior performance of the proposed F-shape-based scheme can be attributed to the use of (i) a highly dense mesh formed on the ROI in the macula region, (ii) atlas registration that puts the F-shapes of all the subjects on a common platform, and (iii) residual thicknesses as input features for the classification.

## 1. Introduction

Multiple sclerosis (MS) is an inflammatory and neurodegenerative disease of the central nervous system (CNS) that usually occurs in young adults and affects twice as many women as men. The neurodegeneration in MS is due to ganglion cell and axonal loss. As a non-invasive imaging tool, magnetic resonance imaging (MRI) can effectively detect inflammation in the nervous system due to MS. However, it is a less accurate modality for detecting axonal and neuronal loss that characterizes MS [1,2,3,4]. MRI is also expensive and is not readily available in most health institutions [5].

The retina contains axons and ganglion cells, which enable us to assess neuroaxonal degeneration. Over the past few years, extensive research has been carried out on the retinal layer to detect axonal loss and neurodegeneration in MS patients using optical coherence tomography (OCT) images [6]. OCT is a new, non-invasive tissue imaging technique that provides cross-sectional images of the retina using infrared light for tissue penetration. In comparison to MRI, OCT is inexpensive and more readily available. Furthermore, in contrast to MRI, it can provide an accurate quantification of neuronal and axonal damage by generating a retinal thickness map which represents the thickness of the layer at each pixel [3,4,7].

Several studies using OCT have shown that the thickness of the peripapillary retinal nerve fiber layer (pRNFL) and macular ganglion cell–inner plexiform layer (GCIPL), which includes both ganglion cell layer (GCL) and inner plexiform layer (IPL), significantly decreased in MS patients [6,8,9]. However, some authors have shown that thinning of GCIPL is a more reliable biomarker to detect axonal loss in MS patients than pRNFL [10,11]. The GCIPL layer is the most affected layer of the macular region in MS patients [10,12,13,14].

Various studies have used predefined regions (sectors) on thickness maps obtained by OCT to quantify neural damage due to MS. For instance, sectors defined by the Early Treatment Diabetic Retinopathy Study (ETDRS) method were used to calculate the average retina layer thicknesses in 9 sectors [15]. Due to averaging over a region, these sectoral methods do not detect the focal spatial details of thickness variations due to MS. Also, these methods rely on an anatomical reference (i.e., center of fovea) to define the sectors [12,16,17].

In other studies, the quantified thickness variations using sectoral-based methods are used to detect subjects having MS using machine learning-based classification methods. Machine learning is a subset of artificial intelligence. In medical contexts, machine learning algorithms provide tools to identify patterns in raw data and use them to diagnose health problems. In particular, machine learning algorithms have been used to detect glaucoma using OCT images [18,19,20] and to detect diabetic retinopathy [21,22]. Machine learning algorithms have also been used to detect MS using OCT images [7,17,23,24,25,26].

Garcia-Martin et al. used an artificial neural network (ANN) classifier with 10-fold cross-validation to distinguish MS subjects from healthy ones by using the mean of peripapillary retina nerve fiber layer (pRNFL) thickness in uniformly divided areas. The input features for the ANN classifier are the calculated mean thicknesses in these areas. They showed that ANN can distinguish MS cases from healthy ones [23].

Cavaliere et al. applied a support vector machine (SVM) classifier to distinguish MS cases from healthy ones. The ETDRS sectoral mean thicknesses in the macula for total retina (between the internal limiting membrane (ILM) and the retinal pigment epithelium boundaries) and choroid layer, and also the temporal–superior–nasal–inferior–temporal (TSNIT) sectoral mean thicknesses in the peripapillary area for total retina, choroid, retinal nerve fiber layer (RNFL), GCIPL, and GCL++ (GCIPL + RNFL) were obtained. Using analysis of area under the curve (AUC), the three most discriminant features were found to be GCL++ thicknesses at the peripapillary region plus retinal thicknesses in the inner nasal and outer nasal sectors in the macula. By using them as input features for an SVM classifier fine-tuned with leave-one-out (LOO) cross-validation, MS can be distinguished from healthy [17].

In another study, using the same dataset as [17], Garcia-Martin et al. defined a grid of 45 × 60 rectangular areas that covers the macular and peripapillary areas, and the thicknesses of RNFL, GCIPL, GCL++, and choroid were measured in that grid. The grid points with high discriminant capability to distinguish MS from healthy are determined for each of those layers by thresholding a distance measure defined between the healthy and MS groups for each grid element. Points exceeding the threshold value are considered inputs for SVM and feedforward neural network classifiers. They showed that GCL++ thickness in the macula using a feedforward neural network classifier has the greatest capability to distinguish MS, and choroidal thickness does not exhibit any discriminatory capacity [26]. Therefore, using a different feature selection approach on the same dataset as [17], it was shown that although both macula and peripapillary regions are scanned by OCT images, the macula region has the most discriminant features [26].

Khodabandeh et al. used the average thicknesses over 20 × 20, 30 × 30 and 40 × 40 pixel square areas of GCIPL and inner nuclear layer (INL) layers in the macula as input features for the MS classifiers. They showed that the square size of 40 × 40, using Linear SVM as a classifier with 10-fold cross-validation, has the best performance. This study utilized separate test data to evaluate the classifiers [7].

Montolio et al. developed a four-layered deep neural network classifier to distinguish MS from healthy using macular GCL in ETDRS sectors. To overcome overfitting, drop-out and 10-fold cross-validation techniques were used [27]. This study only focused on the macular region, and they showed that their result is better than previous studies using both the macula and the peripapillary regions [27].

The classifiers provided in all the studies mentioned above rely on comparing average thicknesses in a local region to distinguish MS subjects from healthy ones. Using pointwise thicknesses instead of the average thicknesses over a local region would enable us to compare the thicknesses more accurately.

An F-shape-based atlas registration method developed by Charlier et al. was used to detect glaucoma using OCT images of the pRNFL retinal layer and a machine learning-based classifier [19]. This classifier was able to detect the disease more accurately than the classifier developed based on the sectoral method because it relies on a pointwise comparison of the thicknesses, which means it utilizes the full spatial details of the OCT images. F-shape considers the retinal surface as a geometry and the retinal layer’s thickness as a signal mapped on the retinal surface. Therefore, the retinal surface, together with retinal layer thickness, is considered a single object called an F-shape [28]. The F-shape-based atlas registration method developed by Charlier et al. uses a reference F-shape for this registration, which is called the mean template. The mean template and registered F-shapes are obtained by solving an optimization problem that minimizes the geometric-functional distance between the mean template and the F-shapes of OCT samples, and at the same time, the dissimilarity between the mean template registered on the F-shape of OCT samples (registered samples) and the F-shape of OCT samples [19].

The objective of this paper is to develop an F-shape-based scheme for the binary classification of MS using the thickness of GCIPL in the macula region of the OCT images. Our motivation for using an F-shape framework for the classification of this disease is driven by the work of Lee et al. [19] in which the F-shape-based atlas registration method, as proposed by Charlier et al. [28], was used to classify another disease, namely, glaucoma, which affects the pRNFL layer of OCT images.

Starting from the F-shapes of GCIPL in the macula region of OCT images of a set of subjects with and without MS, the proposed scheme consists of performing an atlas registration, which results in a set of registered F-shapes corresponding to all the subjects, and an F-shape of the mean template. For each subject, a residual F-shape is obtained by finding the difference between the thickness of the mean template and that of the registered F-shapes of each subject at the corresponding point spread over a grid of the layer. The set of residuals for all the subjects in the training set is used as a feature to train a classifier, and finally, the resulting trained classifier is used to classify whether a given OCT test image is the image of a healthy subject or that of a subject having MS.

This paper is organized as follows: Section 2 briefly introduces the sectoral method and F-shape-based atlas registration method, provides details of the proposed F-shape-based classification scheme using GCIPL thicknesses, and describes the details of the dataset used to validate the proposed scheme. Section 3 provides the experimental results on the proposed F-shape-based scheme for the classification of MS and a comparison with the classification results reported and obtained using sectoral-based methods. Section 4 presents the conclusions.

## 2. Materials and Methods

This section begins with a brief description of the sectoral method for quantifying the thickness of retinal layers in OCT images, since it is the most commonly used method for detecting diseases affecting the thickness of retinal layers. We then explain in detail the F-shape-based atlas registration method, since this method forms the basis of our proposed scheme for detecting MS. An illustration of the application of this method using our data is provided afterwards. Finally, in this section, the proposed F-shape-based classification scheme for detecting MS is developed, along with a brief description of the dataset used to validate our proposed scheme.

### 2.1. ETDRS Sectoral Method

In a sectoral method for quantifying the thickness of retinal layers using OCT images, the retinal layer is divided into sets of areas, and the average thickness of each area is obtained.

**Figure 1:**
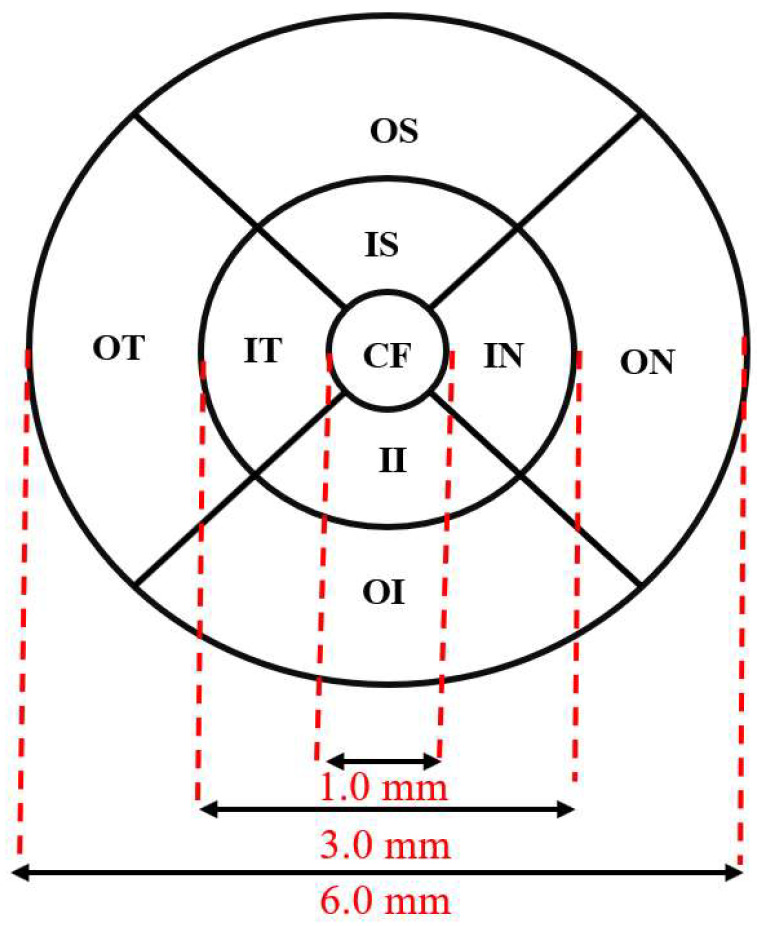
ETDRS grid for thickness analysis in the macula region.

In particular, the ETDRS grid divides the macula region into nine sectors within three concentric circles. The fovea is located in the central 1 mm circle, and the inner and outer rings are 3 mm and 6 mm in diameter, respectively. The inner and outer rings are divided into quadrants by two intersecting lines, as shown in Figure 1. The average thickness of the layer is calculated within each of the nine sectors, which are defined as follows: central fovea (CF), inner superior (IS), inner nasal (IN), inner inferior (II), inner temporal (IT), outer superior (OS), outer nasal (ON), outer inferior (OI) and outer temporal (OT) [15].

### 2.2. F-Shape-Based Atlas Registration

The method of F-shape-based atlas registration was introduced by Charlier et al. in [28], in which a registered F-shape for each of the objects in the set of *N* F-shapes on a common (mean) template is obtained. There are two main steps in this method:

- Generating F-shape objects for all data samples containing a pointwise representation of the geometry of the data samples and a function assigned to each point of their geometry.
- Performing an atlas-based registration on the F-shape objects using a common reference to match the geometries of various data samples.

An F-shape object for the ith subject (1≤i≤N) is denoted by a pair $\left(X^{i},f^{i}\right)$. The geometrical surface of the F-shape is defined by 3D coordinates of the points on the surface of the data sample and is represented by $X^{i}$. The function (signal) part of the object is defined by the values of the function at these points on the surface of the data sample and is represented by $f^{i}$. A crucial step in the F-shape-based atlas registration is obtaining a reference F-shape to be estimated from the given set. It is for this reason, in this F-shape-based registration, this reference F-shape is also called the mean template, denoted by ($X_{m},f_{m})$. The F-shape of the *i*th subject is registered to the mean template, and the resulting F-shape is called the registered F-shape of the *i*th subject and is denoted by $\left({\tilde{X}}^{i},{\tilde{f}}^{i}\right)$. To obtain the mean template and registered F-shapes, an optimization problem that simultaneously minimizes a geometrical–functional distance between the mean template and the F-shape objects generated from our subjects, plus a dissimilarity between the registered F-shapes and the original F-shapes, is formulated [28]. This formulation is referred to as the objective function and should lead to a convergent solution.

#### 2.2.1. Formulation of the F-Shape-Based Atlas Registration Problem

In our study, we choose the objective function *J* given below with an iterative formulation as provided in [29] to carry out the F-shape-based atlas registration:

(1) $$J\left(X_{j},f_{j},p_{j},\zeta_{j}\right)\dot{=}\gamma_{f}.{\left\|f_{j}\right\|}_{L_{2}}^{2}+\sum_{i=1}^{N}\left(H\left(X_{j},p_{j}^{i}\right)+\gamma_{\zeta }{\left\|\zeta_{j}^{i}\right\|}_{L_{2}}^{2}+\gamma_{w}A(\left({\tilde{X}}_{j}^{i},{\tilde{f}}_{j}^{i}\right),\left(X^{i},f^{i}\right)\right)$$

where $\left(X_{j},f_{j}\right)$ denotes the mean template at the *j*th iteration; $p_{j}$ and $\zeta_{j}$ denote, respectively, the sets of geometrical and functional transformation parameters for all the samples in the *j*th iteration, $p_{j}^{i}$ and $\zeta_{j}^{i}$ are, respectively, the sets of geometrical and functional transformation parameters for the *i*th sample in the *j*th iteration, and $\left({\tilde{X}}_{j}^{i},{\tilde{f}}_{j}^{i}\right)$ is the registered F-shape of the *i*th sample at the *j*th iteration. In the objective function given by (1), $H\left(X_{j},p_{j}^{i}\right)$ represents the geodesic distance between the mean template in the *j*th iteration and registered F-shapes, and $A(\left({\tilde{X}}_{j}^{i},{\tilde{f}}_{j}^{i}\right),\left(X^{i},f^{i}\right))$ represents the dissimilarity between the *i*th registered F-shape in the *j*th iteration $\left({\tilde{X}}_{j}^{i},{\tilde{f}}_{j}^{i}\right)$ and the F-shape of the *i*th sample $\left(X^{i},f^{i}\right),$ $\gamma_{f}$, $\gamma_{\xi }$ and $\gamma_{w}$ are positive weighting coefficients providing a balance between the different terms; and ${\left\|x\right\|}_{L_{2}}$ denotes the L2-norm of the vector ***x***. The detailed expressions for the H and A can be found in [28,29]. The objective function given by (1) is non-convex, however, Charlier et al. in [28] have shown the existence of a solution of the F-shape atlas registration problem based on this objective function.

#### 2.2.2. Solution of the F-Shape-Based Atlas Registration Problem

To minimize the objective function, the iterative adaptive gradient descent algorithm is used. As the gradient descent method is used, the mean template and registered F-shapes are iteratively updated through the optimization process [28,29]. Numerically, the iterative process is stopped when the difference between the values of the objective function at two consecutive iterations is less than $10^{-4}$.

### 2.3. An Illustration of the Application of F-Shape-Based Atlas Registration

At the end of the atlas registration process, we have a mean template ($X_{m},f_{m})$ and a set of registered F-shapes for the subjects over a common platform provided by the mean template. The residual thickness of a subject is defined as the offset between the function of the mean template and the function of the registered F-shape for the subject, that is, $f_{m}-{\tilde{f}}^{i}$. The F-shapes of the residual thicknesses are denoted by ($X_{m},f_{m}-{\tilde{f}}^{i})$ for the *i*th subject. Finally, the residual thickness for each of the registered F-shapes is obtained. The set of residuals can be used for the function variability analysis.

**Figure 2:**
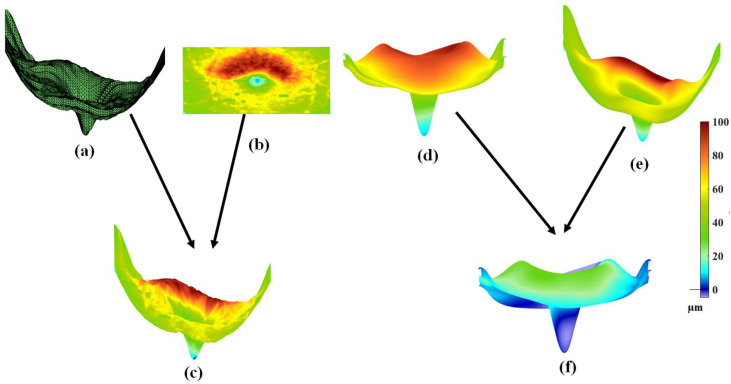
An illustration of the application of the F-shape-based atlas registration method as applied to the GCIPL of an OCT image: (**a**) The ILM surface of the OCT image. (**b**) The thickness map of the GCIPL layer. (**c**) The F-shape of the GCIPL layer obtained by mapping the thickness map given by (**b**) GCIPL on the ILM surface given by (**a**). (**d**) The mean template obtained by carrying out atlas registration on a set of F-shapes, including the F-shape of the subject corresponding to (**c**). (**e**) The registered F-shape corresponding to the F-shape given in (**c**). (**f**) The residual F-shape corresponding to the registered F-shape given in (**e**).

Charlier et al. [28] demonstrated the application of their F-shape-based atlas registration method by applying it to perform atlas registration on the F-shapes of the NFL layers of a set of OCT images. Since in this paper we are concerned with the problem of classifying MS that affects the thickness of GCIPL in the macula region, we illustrate the F-shape-based atlas registration method as described above by applying it to GCIPLs of a set of 72 OCT images of the Isfahan dataset [14]. Figure 2 gives an illustration of the application of this method with respect to a specific subject from the Isfahan dataset. Figure 2a and Figure 2b show the ILM surface (the reference surface) and the thickness map of the GCIPL layer of the subject, respectively. Figure 2c shows the F-shape of the GCIPL layer of this subject resulting from the mapping of the GCIPL layer on the ILM surface. By conducting the atlas registration on the F-shapes of all the subjects in the set, the mean template, as shown in Figure 2d, and the registered F-shape for the subject in question, as shown in Figure 2e, are obtained. Finally, the functional residual of the subject, as shown in Figure 2f, is obtained as the difference between the mean template and the registered F-shape of the subject.

### 2.4. Proposed F-Shape-Based Classification Scheme to Detect MS Using the Residual Thickness of GCIPL

**Figure 3:**
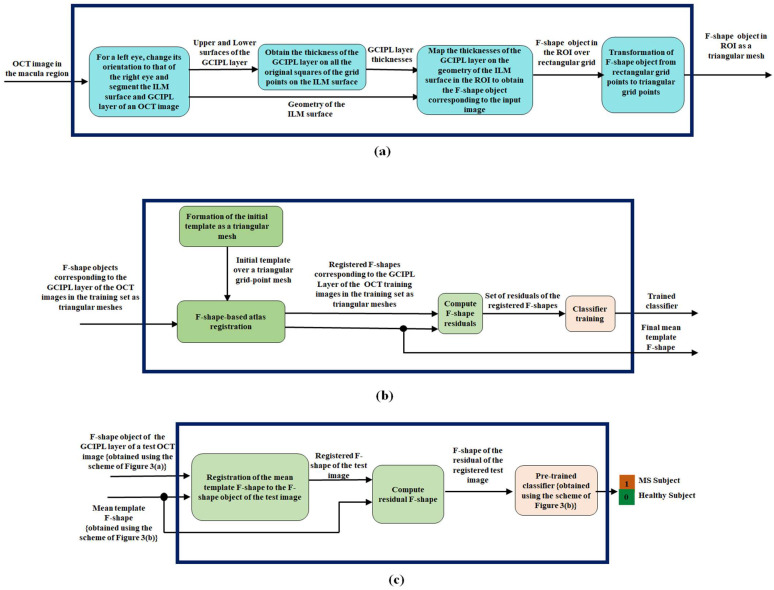
The proposed F-shape-based scheme applied to the GCIPL of an OCT image for the classification of MS: (**a**) The generation of F-shape object for the GCIPL layer of an OCT image. (**b**) Training of a classifier and the generation of the mean template. (**c**) The process of classification of MS.

In this subsection, we present our proposed F-shape-based classification scheme to detect MS. The block diagram of the proposed scheme, having three parts, is shown in Figure 3.

In the first part of the scheme shown in Figure 3a, given an OCT image as input to this part, an F-shape object corresponding to GCIPL of the OCT image is obtained. Note that the orientation of all the left eyes should be changed to that of the right eye. As shown in this figure, this task is accomplished first by finding the geometry of the ILM surface, which is the upper surface of the top layer of the OCT image, which is called RNFL. The geometry of this surface is used as a reference surface. The GCIPL layer is segmented by identifying its top and bottom surfaces. Once the GCIPL layer is segmented, its thicknesses at the various points with reference to the rectangular grid points on the ILM surface are obtained in the third block of Figure 3a by restricting to a region, referred to as a region of interest (ROI), of the ILM surface. Subsequently, the thicknesses of the GCIPL layer are mapped to the geometry of the ILM surface in the ROI at the corresponding points to obtain the F-shape object over the rectangular grid point mesh in the ROI of the GCIPL layer of the input OCT image.

**Figure 4:**
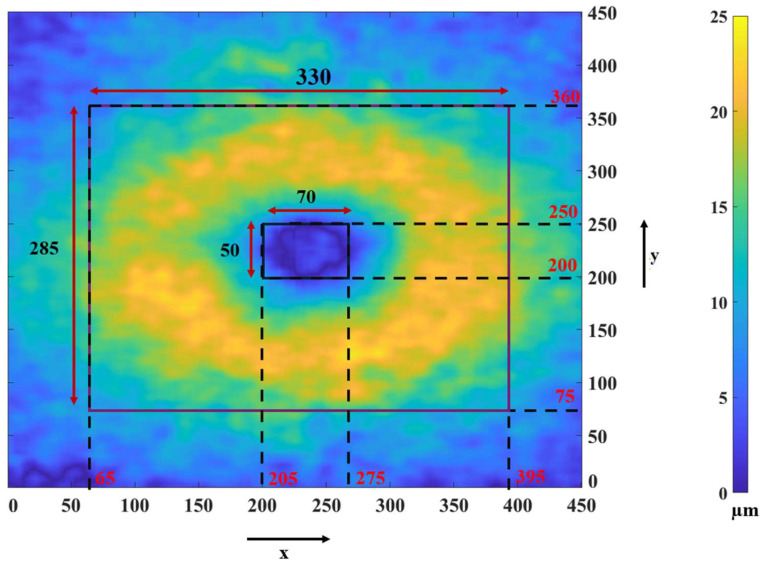
Map of the GCIPL thickness differences between a healthy subject and a subject with MS, averaged over a group of 37 healthy eyes and 35 MS eyes.

Now we describe how we determine the ROI and use it for obtaining the F-shape object for each GCIPL. Researchers have reported that there is a significant difference in the thickness of the macula part of GCIPL of a subject having MS and that of a healthy subject in a horseshoe-like region around the fovea [13,30]. Hence, instead of focusing on the entire macula part of the GCIPL layer, we focus on a region of interest (ROI) in the layer in which the thickness difference between a healthy subject and a diseased subject is significant. In order to find out the ROI for our study, we consider a group of 37 eyes from healthy subjects and a group of 35 eyes from subjects with MS, and obtain the average thicknesses at all the grid points for each group. Figure 4 shows the map of the average thickness differences between the two groups. In this figure, the amounts of thickness reductions in the various regions of the GCIPL layer are indicated by different colors according to the color bar shown on the right side of the figure. The color of the regions varies from the very light yellow color, indicating the most significant thickness difference, to the very dark blue color, indicating little or no thickness difference between the layer of a healthy subject and that of a subject with MS. Based on the observation provided by Figure 4, we should select an ROI that includes most of the yellow regions with the inclusion, as little as possible, of the blue regions. Thus, we select an ROI as a region between two rectangles. The outer rectangle consists of 330 × 285 pixels, ranging from the pixel position 65 to the pixel position 395 along the x-axis and from the pixel position 75 to the pixel position 360 along the y-axis. The inner rectangle consists of 70 × 50 pixels, ranging from the pixel position 205 to the pixel position 275 along the x-axis and from the pixel position 200 to the pixel position 250 along the y-axis.

Note that the F-shape objects created in the third block of Figure 3a are defined over a rectangular mesh with a very large number of grid points dictated by the resolution of the images. If the atlas F-shape registration, training, and the classification of the input image are to be carried out using the values of an F-shape object at all the grid points, it would be computationally very expensive. Hence, finally, in the fourth block of Figure 3a, we select only a small subset of the total number of rectangular grid points from the F-shape object of an image. It has been reported that for a given number of vertices, a triangular mesh can fit the geometry of a surface better than a rectangular mesh can [31]. It has been reported in refs. [32,33] that the use of a regular mesh, such as simple structures like triangles or squares, is computationally less expensive. Accordingly, in the fourth block of Figure 3a, we form the F-shape object of GCIPL over a triangular mesh using only 4% of the rectangular grid points to form a triangular mesh. In order to achieve this, we aim to divide the ROI of the image into squares of equal size and then divide each square by its diagonal. In order to have such a triangular mesh in the ROI, which has a given shape and size, we estimate that the length of each side of the square ought to be 4.92 units, if only 4% of the original grid points have to be used. However, with this length of the sides, the vertices of the square will not necessarily coincide with those of the original rectangular grid points. Thus, for each vertex of the triangular mesh to coincide with the nearest original rectangular grid point, a side of the square needs to be decreased to 4 units or increased to 5 units. This will result in the ROI getting divided into rectangles of four different sizes, namely, rectangles of sizes 4 × 4, 4 × 5, 5 × 4, and 5 × 5. Each of these types of rectangles is divided into two right-angled triangles by its diagonal, thus finally forming an F-shape over a triangular mesh at output of Figure 3a.

Our overall objective is to classify a test image by a classifier as to whether it is the image of a subject having MS or not, using the F-shape of GCIPL of the image. In order to train the classifier and use it for the classification of an arbitrary OCT image, all the images involved in the training and testing of the classifier must first be registered to a common OCT image. Therefore, in the second part, shown in Figure 3b, of the proposed scheme, we first performed a registration of all the training images to a common image called the mean template image. For our scheme, we perform such a registration by using an atlas F-shape registration method [28], in which an iterative registration is performed starting with the F-shapes of the GCIPL layers of all the training images that are obtained by using the scheme Figure 3a on a triangular mesh and the initial F-shape of the mean template, also on a triangular mesh. In order to obtain the initial mean template, we first form a rectangular mesh using the same geometry for its ROI and the same rectangular resolution as that used for the images, with the thickness value at each rectangular grid point being zero. This initial F-shape of the mean template over the rectangular mesh is then transformed into a triangular mesh using the same procedure as that used in Figure 3a for forming the triangular mesh of the images, but this time using 4.5% rather than 4% of the original rectangular grid points. The reason for choosing a higher number of grid points for forming the initial mean template is that this choice enables us to perform the registration of the F-shape objects of the individual subjects on the mean template with a more detailed alignment [28].

After the completion of the atlas registration, we have a set of registered F-shapes of all the GCIPL layers of the OCT training images in the training set and the F-shape of the final mean template, each over a common triangular mesh. Next, in the second part of the proposed scheme, the set of registered F-shapes of the training and the F-shape of the final mean template are used by the F-shape residual formation block to compute the F-shape residuals for each subject as the offset between the thickness of the registered F-shape of an individual subject and the mean template. Finally, the set of residuals of all the training images is used as a set of features to train a classifier. Hence, the output of the second part of the proposed scheme is a trained classifier and the F-shape of the final mean template.

Finally, in the third part of the proposed scheme shown in Figure 3c, we classify a subject as a healthy subject or a subject having MS. Before this task is initiated by this part of the proposed scheme, the F-shape of the subject image is first obtained over the same triangular mesh as that used for the training images by using the scheme of Figure 3a. This F-shape of GCIPL of the subject, along with the F-shape of the mean template (obtained from Figure 3b), is used as the input to the third part of the proposed scheme, in which a registration of the F-shape of the subject to the F-shape of the mean template is performed by its first block. The resulting F-shape of the test subject and the mean template are then used by the second block of this part to obtain the residual F-shape of the registered test image. Finally, this residual F-shape is used by the trained classifier in the last block of Figure 3c for a binary classification of the subject for MS.

### 2.5. OCT Image Dataset Used in the Proposed Scheme

The OCT images used in this study are from the dataset referred to as the Isfahan dataset [14]. The images in this dataset were collected at the Kashani Comprehensive MS Center of Isfahan University of Medical Science. The images in this dataset were acquired using Spectralis SD-OCT hardware along with the Heidelberg Eye Explorer (HEYEX) version 5.1 (Heidelberg Engineering, Heidelberg, Germany) [14]. The scanning area is 6 mm by 6 mm, focusing on the fovea region. This dataset contains a total of 38 subjects (30 females and 8 males). The gender demography of the participants is provided in Table 1. From the total of 38 subjects, 20 subjects constitute the group of healthy subjects, and the remaining 18 constitute the group of relapsing–remitting (RR) MS subjects. From the 18 MS subjects, the scans of 35 eyes have been obtained. Similarly, from the 20 healthy subjects, the scans of 37 eyes have been obtained.

We randomly select 26 eyes with MS and 28 healthy eyes to form the training set. The remaining eyes from both groups are used for testing.

## 3. Experimental Results and Discussion

As stated earlier in Section 1, the classification of MS using GCIPL of OCT images has been carried out in [7,17,26] by analyzing the thickness of the layer in the macula region or the macula and peripapillary regions together by partitioning such a layer into sectors and using the average thickness of these sectors. It has also been pointed out in the literature [26,27] that the classification of the disease using only the macula region leads to a superior classification result than that using the macula and peripapillary regions together. Accordingly, in this section, we apply our proposed F-shape framework to classify MS using the thickness of the GCIPL layer only in the macula region. This section is organized as follows: In Section 3.1, the average thicknesses of the retinal layers for all the points in the various layers are obtained for each subject, and these averages are compared between the MS and healthy groups using *p*-values. Moreover, in this subsection, the pointwise average thickness maps of the retinal layers are provided for the healthy and MS groups. Section 3.2 describes the choice of classifier used and its details for the detection of MS. In Section 3.3, the classification results of detecting MS using the proposed F-shape-based scheme are presented and compared with those of the methods in the literature that use sectoral schemes and SVM classifiers for the detection of the disease.

### 3.1. Thickness Map of Retinal Layers

**Figure 5:**
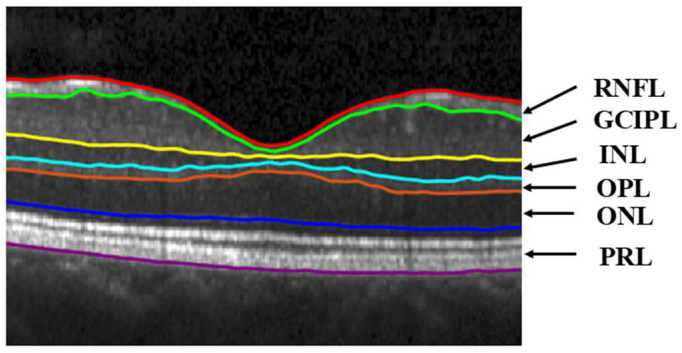
A schematic of the segmented layers of the OCT image of a subject.

A schematic showing the retinal layers, that is, RNFL, GCIPL, INL, outer plexiform layer (OPL), outer nuclear layer (ONL), and photoreceptor layer (PRL), of an OCT image is given in Figure 5. The average thickness and standard deviation (SD) of all these layers for the healthy and MS subjects of our dataset are provided in Table 2. This table also lists the *p*-values by applying the *t*-test between the average layer thicknesses of healthy and MS groups.

The *p*-values in Table 2 show that the average thicknesses of GCIPL and RNFL in the macula are significantly affected by MS, while the other layers are not significantly affected. However, the statistical significance of the differences between the healthy and MS groups is considerably higher for GCIPL, which is in accordance with what is reported in the literature that the GCIPL layer is the most-affected layer in the macula region by MS [10,12,13,14]. For these reasons and the fact that results using the GCIPL layer have already been reported [7,17,26,27], we choose this layer in this paper to study the effect of MS so that we can compare our results with those reported in the literature.

**Figure 6:**
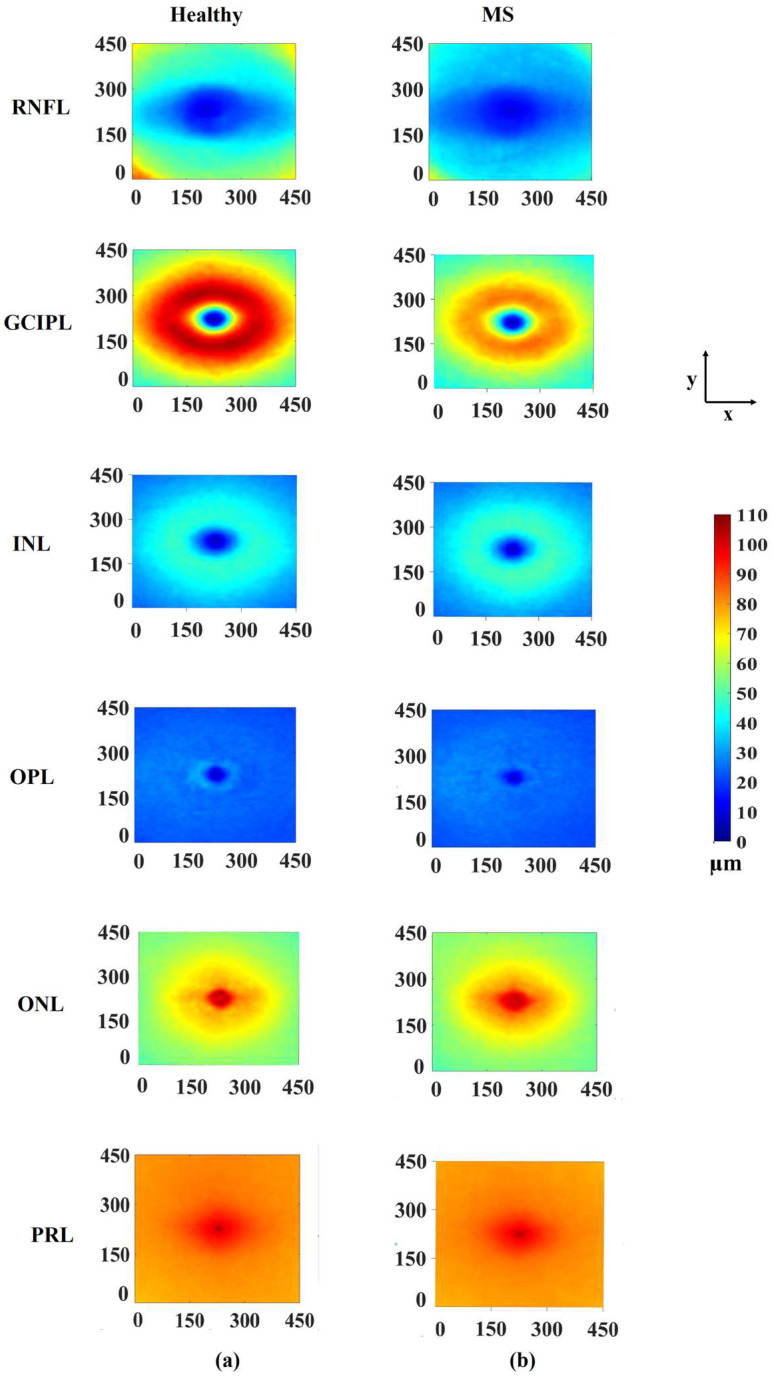
Average thickness maps of MS and healthy subjects for various retinal layers: (**a**) healthy group and (**b**) MS group.

Figure 6a shows, for the various retinal layers, the pointwise average thicknesses across the various points of the subjects in the healthy group, and Figure 6b shows the same for the MS group. It is seen from these figures that the average thicknesses at the various points in the horseshoe region for the subjects in the MS group get significantly reduced from those in the group of healthy subjects for GCIPL. For this layer, the values of average thicknesses for the various points vary between 2.8 µm and 106.1 µm for the healthy subjects, whereas thicknesses vary between 4 µm and 85.4 µm for MS subjects. Thus, the range of thickness variations in the GCIPL layer in the group of MS subjects is smaller than that in the group of healthy subjects.

### 3.2. Classifier Used for the Detection of MS 

Since the SVM classifier has been used to detect MS by the schemes in the literature [7,17], we compare our proposed scheme with these schemes using the same classifier. In our work for the binary classification of MS, the residuals of the registered F-shapes are used as input features to the classifiers. Since the residual of each image is defined over 4120 triangular grid points, the total number of inputs to the classifier is equal to this number. For the classifiers based on the sectoral method, the number of inputs to the classifier is nine, that is, the same as the number of sectors used in that method. Therefore, the number of inputs to the classifiers used in the ETDRS method is nine, where each of the inputs is equal to the average thickness of one of the nine sectors.

In order to avoid overfitting and improve the robustness of the SVM classifiers used in our study, we use the leave-one-out (LOO) grid search cross-validation method [34]. In order to perform grid search cross-validation, we first split the training data into k folds. Then, for every unique combination of hyperparameters in the grid, the classifier is trained k times. In each iteration, one fold is used as a validation set and the remaining k-1 folds are used for training. At the end of each iteration, we have one trained model of the classifier, which is used to get the classification score for each of the images in the validation set of that iteration. An average score is obtained over all the images in the validation set of that iteration. These average scores from all the *k* iterations are averaged to obtain the cross-validation (CV) score for that specific set of hyperparameters. Then, the particular combination of the hyperparameters that produce the best (highest) CV score is used to train the classifier on the entire training set. The classifier thus trained is the final trained model.

In our study, the size of the training set is 54 samples (26 MS + 28 healthy), and we also use *k* = 54 as the number of folds for the grid search cross-validation. Since in our cross-validation, *k* is chosen to be equal to the number of images in the training set, this cross-validation method is also referred to as leave-one-out (LOO) cross-validation [34]. Table 3 gives the values of the hyperparameters of the selected trained classifier, which corresponds to the highest CV score. The fact that using 54 OCT images from the Isfahan dataset as a training set provides a value of 95% for the highest CV score, and that we have used a grid search cross-validation technique, ensures that we have obtained a well-trained model that should generalize to new data (test set) [35].

#### Support Vector Machine (SVM) Classifier

An SVM classifier is a supervised learning classifier that separates two classes in a binary classification by maximizing the margin between the two classes and by finding an optimal hyperplane that separates the two classes [36,37]. If the two classes are not separable by a hyperplane, a kernel function is used to map the data into a higher-dimensional feature space so that a hyperplane in the higher-dimensional space is constructed that separates the data into two classes. There are four common types of kernel functions that are used for SVM classifiers [38]:

Linear kernel function:

(2) $$K\left(X_{i},X_{j}\right)={X_{i}}^{T}X_{j}$$

Polynomial kernel function:

(3) $$K\left(X_{i},X_{j}\right)={{(\gamma X_{i}}^{T}X_{j}+r)}^{d},\gamma >0$$

Radial basis function (RBF):

(4) $$K\left(X_{i},X_{j}\right)=\exp\left(\gamma {\left\|X_{i}-X_{j}\right\|}^{2}\right),\gamma >0$$

Sigmoid kernel function:

(5) $$K\left(X_{i},X_{j}\right)=tanh{(\gamma X_{i}}^{T}X_{j}+r)$$

where $X_{i}$ and $X_{j}$ are two vectors from the feature space, γ is a parameter that determines the shape of the decision boundary for the polynomial, RBF and sigmoid functions; r is a constant employed to shift the position of the hyperplane obtained using the polynomial and sigmoid functions, and d determines the order of the polynomial in the polynomial kernel.

For an SVM classifier, another hyperparameter, C, called the regularization parameter, is used to control the generalization performance of the SVM classifier [39]. However, the use of a large value of C also increases the classifier complexity. Hence, the value of C is chosen to balance model complexity and generalization performance.

It has been reported in the literature that the SVM classifier is suitable for the analysis of datasets with a small sample size [40,41]. Since the Isfahan dataset in our study is not a particularly large dataset, we use SVM for the detection and classification of MS. We have a total of 54 training images, and for each image we have a total of 4120 vertices in the triangular mesh; the size of the input data for our proposed scheme is 54 × 4120. This is a very large data size for an SVM classifier, and hence, the dimensionality of this input data must be reduced. We reduce the size of the input data to 54 × *k* by applying principal component analysis (PCA) as a feature reduction method to our input data of size 54 × 4120. The value of *k* is the number of the most significant eigenvalues of the matrix representing the covariance of the input data matrix. The PCA method guarantees a reduction in the dimension of the input data of size *m × n* (*m* < *n*) to be at least *m* × *m* when all the m eigenvalues of the covariance matrix of the input data matrix are used in PCA. The scree plot [42] can be used to determine whether the size of the input data can be reduced to *m* × *k*, where *k* < *m*. A scree plot is a graph of $\sum_{i=1}^{k}\lambda_{i}/\sum_{i=1}^{m}\lambda_{i}$ versus *k* (*k* ϵ [1, *m*]), where (λ_1_, λ_2_, …, λ_m_) is the set of the eigenvalues of the covariance matrix of the input data arranged in a decreasing order of magnitude. Note that all the eigenvalues of the covariance matrix, which is symmetric with non-negative diagonal elements, are non-negative. The optimum number of *k* is determined by finding the point at which the cumulative scree plot flattens out. For our input data matrix, the optimum value of *k* is found to be 10. The reduced data matrix is obtained by post-multiplying the input data matrix *A* by ${V_{k}}^{T}$, where $V_{k}$ is a submatrix consisting of the first *k* columns of the right singular matrix *V* resulting from the PCA of *A*. Thus, in our proposed scheme, the dimension of *A* gets reduced from 54 × 4120 to 54 × 10.

For the sectoral method, the dimension of the input data is 54 × 9, 54 being the total number of training images and 9 being the number of sectors. For this scheme, the scree plot reveals that even the use of all the 9 eigenvalues of the data covariance matrix does not flatten the scree curve, and hence, it is not necessary to carry out the PCA for the dimension reduction in the input data.

Table 3 provides the kernel functions, the values of the corresponding underlying parameters, and the best CV score, as determined by the LOO grid search algorithm [34] when SVM classifiers are used for our proposed scheme and sectoral method.

### 3.3. Results, Comparison, and Discussion

In this section, the performance of the proposed F-shape-based method is compared with that of sectoral-based methods on an SVM classifier. The performance of the methods is determined in terms of the classification to distinguish between healthy and multiple sclerosis subjects. The common performance metrics are accuracy (ACC), sensitivity (SEN), specificity (SPE), precision (PREC), F1-score (*F*1), and the area under the curve (AUC). The metrics ACC, SEN, SPE, and PREC are defined as follows:

(6) $$ACC=\frac{TP+TN}{TP+TN+FP+FN}$$

(7) $$SEN=\frac{TP}{TP+FN}$$

(8) $$SPE=\frac{TN}{TN+FP}$$

(9) $$PREC=\frac{TP}{TP+FP}$$

(10) $$F1=2\times \frac{PREC\times SEN}{PREC+SEN}$$

where TP, TN, FN, and FP denote, respectively, true positive, true negative, false negative, and false positive. The metric AUC represents the area under the receiving operating characteristic (ROC) curve formed by plotting SEN as a function of the false positive rate (FPR), defined as

(11) $$FPR=\frac{FP}{FP+TN}$$

The metric AUC is a measure of the trade-off between sensitivity and specificity.

Table 4 gives the performance of different methods of detecting MS, as provided by an SVM classifier, in terms of classification using the four metrics as defined above. The first row of this table provides the results of the square-grid sectoral method, as proposed by Khodabandeh et al. [7] when the SVM classifier is trained on the Charite dataset and tested on the Isfahan dataset [14]. It should be mentioned that the Charité dataset was collected in the NeuroCure Clinical Research Center (NCRC) at Charité—Universitätsmedizin Berlin, Germany. The results in the second row are obtained using ETDRS and TSNIT sectoral-based methods, as proposed by Chew et al. [15] and Garway-Heath et al. [43], and applied to the macula and the prepapillary regions of the OCT images, respectively, using the Miguel Servet Dataset (MSD), a dataset collected in the Miguel Servet Hospital, Zaragoza, Spain [17]. The results of our F-shape-based proposed scheme, when the Isfahan dataset is used to train and test the SVM classifier, are given in the fourth row of Table 4.

It is seen from the results in the first and second rows of Table 4 that the sectoral method [17] provides a performance by focusing on two different regions of the OCT images, better than that of [7], which focuses only on the macula region. However, the performance of the proposed F-shape-based scheme, given in the fourth row of the table, is able to provide a significantly superior performance than the sectoral-based method of [17], despite focusing only on the macula region.

It is seen from the results of the first, second, and fourth rows of Table 4 that there is no uniformity between the datasets used for testing and training of the classifiers. Therefore, in order to make a direct and fair comparison between the proposed scheme for detecting MS with a sectoral-based method, we have chosen the ETDRS sectoral-based method [15] and applied it to detect the MS subjects, when the Isfahan dataset, the same dataset that we have used in our proposed scheme, is used to train and test the SVM classifier. The results of this sectoral-based method are provided in the third row of Table 4. It is clear from the results of the last two rows of this table that the proposed scheme significantly outperforms the scheme of [15].

The significantly higher performance provided by the proposed F-shape-based scheme can be attributed to the use of residual thicknesses of GCIPL as the classification features that are spread over a highly dense triangular grid and the uniformity of the images achieved from the atlas registration of the image of each subject with a common mean template. This characteristic of the proposed scheme is in sharp contrast to a sectoral-based method, in which the average thicknesses of each of a small number of sectors, into which the GCIPL layer of an OCT image is divided, are used as the features for classification.

In our proposed F-shape framework for the detection of MS, the residual F-shape thickness has been used as a feature. Finally, we would now like to compare the discriminative power of such features with that of using the actual thickness as a feature. For this purpose, we perform *t*-tests between the MS and healthy groups by using the residual values of the thickness of GCIPL, as well as using the actual thickness values. The *t*-test result using residual values shows that 91% of the points have significant *p*-values (*p* < 0.05), while that using the actual thickness values shows that only 83% of the points have significant *p*-values. This result shows the advantage of using residual thicknesses as input features over using the actual thicknesses of the layer.

## 4. Conclusions

In this paper, an F-shape-based scheme has been developed for the classification of MS, which results in variability in the thickness of GCIPL between healthy subjects and those with MS. The proposed scheme consists of first representing the thickness of the GCIPL layers in the macula region of OCT images for a set of healthy and diseased subjects as F-shape objects, and then performing an atlas registration. The residual F-shapes, defined as the difference between the F-shape of the mean template and the individual registered F-shapes, are finally used as features to train an SVM classifier, which is used to classify the individual subjects accordingly.

The performance of the proposed F-shape-based scheme for the classification of MS is compared with that of a couple of sectoral-based schemes for the classification of the disease. Since these two schemes did not use the Isfahan dataset, which we use for our proposed scheme, we could not di-rectly compare our proposed scheme with these two schemes. It is for this reason that we have also trained and tested the ETDRS sectoral method using the Isfahan dataset, so that we could directly compare our proposed scheme with this ETDRS sectoral method. The proposed F-shape-based scheme has been shown to significantly outperform the sectoral-based schemes, regardless of the dataset used for training and testing.

The ability of the proposed F-shape-based scheme for classification to provide the performance superior to that of the sectoral-based scheme can be attributed to a number of factors: (i) the proposed scheme is applied to a region of interest of GCIPL whose thickness is more affected by MS, (ii) the classification in the proposed scheme is carried out by using the residual thicknesses of the subjects as features instead of the thicknesses of the GCIPL layer of the OCT image, as used by the sectoral-based method, (iii) the use of a very large number of features in the proposed scheme as compared to the use of a small number of features corresponding to the number of sectors used in the sectoral-based methods, (iv) the use of a common platform furnished to all the training and testing images by the use of atlas registration in the F-shape-based proposed scheme.

## Figures and Tables

**Table 1:** Gender breakdown of participants.

Group	Number of Subjects	Number of Eyes
MS	18 (16 F/2 M)	35
Healthy	20 (14 F/6 M)	37

**Table 2:** Comparing average retinal layer thickness between healthy and MS subjects.

Retinal	Healthy *N* = 37	MS *N* = 35	
	Mean, µm (SD)	Mean, µm (SD)	*p*-Value
RNFL	38.3 (4.1)	31.8 (4.6)	1.47 × 10^−8^
GCIPL	75.9 (6.2)	62.9 (5.4)	3.07 × 10^−14^
INL	37.0 (2.8)	37.5 (2.7)	0.44
OPL	24.2 (1.3)	24.4 (1.1)	0.53
ONL	63.6 (5.6)	63.7 (5.7)	0.94
PRL	82.1 (2.6)	81.8 (2.5)	0.61

**Table 3:** Kernel functions, their underlying hyperparameters, and best CV score as determined by the LOO grid search algorithm [34] for the SVM classifiers.

Classifier	Kernel Function	γ	C	r	Best (Highest) CV Score
SVM classifier using the proposed scheme	linear	0.001	40	0	95%
SVM classifier using the sectoral-based method	linear	0.1	0.4	0	91%

**Table 4:** Performance evaluation of different methods to distinguish between healthy and multiple sclerosis subjects using an SVM classifier.

Method	Dataset Used	Performance
Square-grid sectoral method of Khodabandeh et al. [7] (2023)	Charite dataset for training and Isfahan dataset [14] for testing	ACC = 88% SEN = 88% PREC = 89% F1 = 84%
Sectoral method of Cavaliere et al. [17] (2019), which uses a combination of the ETDRS method of [15] when applied to the macula region and the TSNIT method of [43] when applied to the optic nerve region	Miguel Servet dataset [17] for training and validation using leave-one-out cross-validation	ACC = 91% SEN = 89% SPE = 92% AUC = 97%
ETDRS sectoral-based method of [15]	Isfahan dataset [14] for training and testing	ACC = 89% SEN = 89% SPE = 89% AUC = 94%
Proposed F-shape-based method	Isfahan dataset [14] for training and testing	ACC = 95% SEN = 89% SPE = 100% AUC = 99%

## Data Availability

The Isfahan dataset used in the current study is not publicly available. Any inquiry concerning this dataset may be directed to Professor Raheleh Kafieh.

## Abbreviations

The following abbreviations are used in this manuscript:

ACC	Accuracy
ANN	Artificial neural network
AUC	Area under the curve
CF	Central fovea
CNS	Central nervous system
CV	Cross-validation
ETDRS	Early treatment diabetic retinopathy study
F-shape	Functional shape
F1	F1-score
GCIPL	Ganglion cell–inner plexiform layer
GCL	Ganglion cell layer
HEYEX	Heidelberg eye explorer
ILM	Internal limiting membrane
IN	Inner nasal
INL	Inner nuclear layer
IPL	Inner plexiform layer
IS	Inner superior
IT	Inner temporal
LOO	Leave-one-out
MRI	Magnetic resonance imaging
MS	Multiple sclerosis
OCT	Optical coherence tomography
OI	Outer inferior
ON	Outer nasal
ONL	Outer nuclear layer
OPL	Outer plexiform layer
OS	Outer superior
OT	Outer temporal
PREC	Precision
PRL	Photoreceptor layer
pRNFL	Peripapillary retinal nerve fiber layer
RNFL	Retinal nerve fiber layer
ROI	Region of interest
RR	Relapsing–remitting
SD	Standard deviation
SEN	Sensitivity
SPE	Specifity
SVM	Support vector machine
TSNIT	Temporal–superior–nasal–inferior–temporal
